# Intelligence and executive functioning in adolescence: comparing autism spectrum disorder and typical development

**DOI:** 10.3389/fpsyg.2025.1733356

**Published:** 2026-01-07

**Authors:** Monika Pudło, Krystyna Rymarczyk, Anna Starowicz, Ewa Pisula

**Affiliations:** 1 Faculty of Psychology, SWPS University, Warsaw, Poland; 2 Faculty of Psychology, University of Warsaw, Warsaw, Poland

**Keywords:** autism spectrum disorder, executive functions, IQ, perceptual reasoning, adolescents, sex differences

## Abstract

**Objective:**

This study examined the relationship between intelligence and executive functions (EF) in adolescents with Autism Spectrum Disorder without intellectual disability (ASD-WID), focusing on the roles of IQ level, sex differences, and comparisons with typically developing (TD) peers matched for age and IQ.

**Methods:**

A total of 214 participants (118 ASD, 96 TD; aged 12–18 years) were assessed with the Wisconsin Card Sorting Test (WCST) and the Color Trails Test (CTT) as measures of planning, cognitive flexibility, and attentional switching. Cognitive ability was assessed using the WISC-R or WAIS-R, yielding full-scale IQ and three cognitive factors: Verbal Comprehension, Perceptual Reasoning, and Working Memory/Resistance to Distractors. Four subgroups (ASD/TD × high/low IQ) were created. Because multiple variables deviated from normality, non-parametric statistics were applied, including Mann–Whitney U tests for group comparisons and Spearman’s rho correlations for associations between IQ indices and EF measures.

**Results:**

No overall EF differences were found between the ASD and TD groups when matched for age and full-scale IQ. Within the ASD group, higher IQ was associated with better planning and cognitive flexibility on the WCST, but not with attentional switching on the CTT; the same pattern appeared in TD adolescents. High-IQ ASD and high-IQ TD adolescents performed comparably on EF measures, suggesting possible compensatory mechanisms in ASD-WID. In contrast, ASD adolescents with lower IQ showed more perseverative errors than TD peers with similar IQ. Across the entire sample (ASD + TD combined), boys scored higher in perceptual reasoning than girls; however, no sex differences were found when analyses were conducted within the ASD group alone, indicating that the observed effect of sex was driven by the TD subgroup rather than by adolescents with ASD. Perceptual reasoning and non-verbal IQ were the strongest correlates of EF performance.

**Conclusion:**

Intelligence—especially perceptual reasoning—plays a key role in EF outcomes in adolescents with and without ASD. High IQ may buffer EF difficulties in ASD-WID, whereas lower IQ is linked to greater executive control difficulties. These findings highlight the need for assessment and interventions tailored to individual cognitive profiles, rather than diagnostic status alone.

## Introduction

Autism Spectrum Disorder (ASD) is a complex neurodevelopmental condition characterized by persistent deficits in social communication and interaction, along with restricted and repetitive patterns of behavior, interests, or activities (American Psychiatric Association, 2022). These core symptoms can vary in intensity and expression across individuals and developmental stages, reflecting the substantial heterogeneity within the autism spectrum. According to the DSM-5 and ICD-11, this variability is also shaped by the presence or absence of co-occurring intellectual disability, which has important implications for cognitive functioning and prognosis. This study focuses on individuals with ASD without intellectual disabilities (ASD-WID), i.e., those with an IQ above 70 (American Psychiatric Association, 2013).

Intelligence has long been recognized as a major predictor of adaptive functioning, educational success, and future independence, both in neurotypical and neurodivergent populations (Duncan and Bishop, 2015). Despite its central importance, intelligence is not a unitary or universally agreed-upon construct. In psychological research, it is most commonly defined as a set of cognitive abilities enabling reasoning, problem solving, learning, and adaptation to the environment (Gottfredson, 1997). However, substantial variability exists in how intelligence is conceptualized and operationalized across theoretical frameworks. Classical psychometric approaches emphasize a hierarchical structure with a general factor (g) at the top, reflecting shared variance across diverse cognitive tasks, and more specific abilities such as verbal, visuospatial, or processing-speed domains beneath it (Carroll, 1997). By contrast, the Cattell–Horn model distinguishes fluid intelligence (Gf), associated with novel problem solving and reasoning under minimal cultural influence, from crystallized intelligence (Gc), reflecting accumulated knowledge and language-based skills (Cattell and Horn, 1978). These distinctions are widely used but remain debated, particularly regarding the extent to which Gf and Gc represent separable abilities or different manifestations of the general factor. More recent neurocognitive approaches conceptualize intelligence not as a stable trait but as an emergent property of distributed brain networks, especially frontoparietal circuits responsible for cognitive control, working memory, and attentional regulation (Jung and Haier, 2007). This perspective highlights the dynamic interaction between neural efficiency, network connectivity, and experiential factors, challenging the assumption that intelligence can be fully captured by traditional test scores. Consequently, the operationalization of intelligence varies widely across empirical studies. Some rely on composite IQ scores (FSIQ), others focus on domain-specific indices such as verbal comprehension or perceptual reasoning, while still others use tasks purported to measure fluid reasoning or working memory. These measures are related but not interchangeable; each captures only a subset of the broader construct. Among adolescents with ASD without intellectual disability (ASD-WID), emerging evidence indicates that those with higher IQs—particularly in the above-average range—tend to perform better on tasks that challenge executive functions, such as goal planning, working memory, and cognitive flexibility (Alloway and Alloway, 2009; Cruz et al., 2022). This suggests that intelligence may supports EF performance in ASD, partially mitigating the impact of core deficits. Furthermore, higher non-verbal IQ—often assessed through perceptual reasoning tasks—appears especially relevant in this context. Adolescents with ASD who possess relatively strong visuospatial skills may be more likely to succeed on EF tasks that require strategic processing, abstract reasoning, or rule shifting. Studies indicate that these individuals may utilize visual processing as a compensatory mechanism, bypassing linguistic or social-cognitive demands that are often impaired in ASD (Mottron et al., 2006; Shah and Frith, 1993). However, the compensatory visual-processing hypothesis is not universally supported, and the evidence remains mixed. Enhanced visuospatial performance in ASD appears highly task-dependent and does not reliably generalize to broader EF (Muth et al., 2014). Some studies report that detail-focused strengths may coexist with difficulties in global integration, which can impair performance on complex EF tasks (Happé and Frith, 2006). Moreover, individuals with ASD do not consistently outperform neurotypical peers once task complexity, memory load, or motor demands are controlled (Soulières et al., 2011), suggesting that visual strengths may reflect specific perceptual biases rather than a universal compensatory mechanism. Conversely, adolescents with lower IQ scores—though still within the normative range—may experience difficulties in planning and flexibility due to weaker foundational cognitive resources or limited access to such compensatory strategies (Merchan-Naranjo et al., 2016).

To interpret such within-group variability, it is useful to apply a broader hierarchical framework, such as the CHC model, which groups domain-level abilities relevant to EF without reintroducing the earlier detailed distinctions. Research shows that these cognitive abilities interact dynamically with executive processes such as shifting, inhibition, and updating (Jewsbury et al., 2016). Converging evidence from behavioral and neuroimaging studies has consistently demonstrated the close functional integration between intelligence and EF (Jung and Haier, 2007; Menon and D’Esposito, 2022). Among EF components, working memory updating has been identified as the most robust predictor of IQ (Friedman et al., 2006), while shifting and inhibition also contribute significantly to problem solving through their reciprocal connections with working memory and reasoning. These executive processes are supported by the coordinated activity of prefrontal brain networks—particularly the frontoparietal control network—and their connectivity with subcortical structures such as the amygdala, putamen, and caudate (Kostović et al., 2008). Although intelligence tests are traditionally used to identify high cognitive ability, several studies suggest that executive functions—especially planning, cognitive flexibility, and working-memory control—may better differentiate high-performing or gifted children than IQ alone, as these skills are more directly involved in managing complex, novel, and self-directed tasks (Arffa, 2007; Kane and Engle, 2002). This perspective highlights that EF contributes uniquely to advanced problem solving and creative reasoning, but the evidence remains mixed, as not all studies replicate these effects once socioeconomic or educational factors are controlled (Lozano-Blasco et al., 2022). EF is also crucial for school readiness, math skills, and social adaptation (Munakata and Michaelson, 2021).

In ASD, deficits in executive functioning are commonly reported, especially in the domains of cognitive flexibility and strategic planning (Demetriou et al., 2018; Lage et al., 2024; O'Hearn et al., 2008). The Wisconsin Card Sorting Test (WCST), used in the present study, is particularly effective in identifying such deficits, including perseverative responses and reduced planning efficacy (Heaton et al., 2008). However, these impairments are not universal and may depend on individual cognitive profiles, particularly IQ composition.

Importantly, the relationship between intelligence and executive functioning may also be influenced by sex. Although the literature on sex differences in ASD has been historically limited due to male-biased samples, recent studies suggest that girls and boys with ASD may differ meaningfully in cognitive strengths and weaknesses (Lai et al., 2015; Napolitano et al., 2022). Some evidence suggests that girls with ASD perform better in cognitive flexibility tasks (Bölte et al., 2011), whereas boys may outperform girls in visuospatial reasoning (de Giambattista et al., 2021). Additionally, girls with ASD and IQ above 70 may have higher verbal IQ than boys (Giambattista et al., 2021) and fewer language-related impairments (Saure et al., 2023). These patterns are relevant for understanding the results of the present study, which revealed differences in perceptual reasoning between sexes, but not in verbal or memory components. However, findings on sex differences in cognitive profiles in ASD remain inconsistent. Several studies report minimal or no sex differences in executive functioning once IQ, age, and symptom severity are controlled (Hull et al., 2017; Lehnhardt et al., 2016). Meta-analytic evidence also suggests that verbal advantages in autistic girls are small and not consistently replicated across samples (Mandy et al., 2012). Moreover, some studies indicate that apparent sex differences may reflect diagnostic biases or differential socialization rather than inherent cognitive differences (Ratto et al., 2018). Therefore, while certain patterns emerge in the literature, sex-related cognitive differences in ASD should be interpreted cautiously, given substantial heterogeneity and mixed empirical evidence.

Despite substantial research on intelligence and executive functioning in ASD, important gaps remain. First, few studies have simultaneously examined multiple components of intelligence—including verbal comprehension, perceptual reasoning, and working memory—in relation to EF within the same ASD sample, limiting our understanding of how distinct cognitive profiles shape executive performance (Alloway and Alloway, 2009; Jewsbury et al., 2016). Second, findings are inconsistent regarding whether higher non-verbal or perceptual abilities compensate for EF difficulties, reflecting mixed evidence and methodological heterogeneity (Muth et al., 2014; Soulières et al., 2011; Stevenson et al., 2018). Third, the role of sex is still unclear, with studies reporting conflicting patterns and often lacking direct comparisons between boys and girls matched for age and IQ (Hull et al., 2017; Mandy et al., 2012; Ratto et al., 2018). Finally, most previous research examines ASD or typically developing (TD) groups in isolation, leaving open the question of whether associations between intelligence and EF differ across diagnostic status when groups are matched on cognitive ability (Demetriou et al., 2018; O'Hearn et al., 2008).

The present study addresses these gaps by providing an integrated analysis of intelligence profiles, EF performance, and sex-related variation in a large sample of adolescents with and without ASD, matched for age and IQ. This approach allows us to clarify whether specific cognitive strengths (e.g., perceptual reasoning) are differentially linked to EF across diagnostic groups, and whether sex moderates these associations. The expected theoretical contribution lies in refining our understanding of how intelligence and EF interact in ASD (Jung and Haier, 2007; Menon and D’Esposito, 2022), while the practical contribution concerns identifying cognitive markers that may support individualized educational planning and targeted interventions for adolescents with ASD without intellectual disability (Munakata and Michaelson, 2021).

In line with these aims, the present study examined the relationship between intelligence and executive functions in adolescents with Autism Spectrum Disorder without intellectual disability (ASD-WID), considering the potential moderating roles of IQ level and sex. Specifically, we investigated whether adolescents with different IQ levels within the ASD group vary in their performance on EF tasks assessing cognitive flexibility, planning, and attention switching, and whether these patterns differ from those observed in neurotypical peers matched for age and IQ. We also explored whether the relationship between intelligence and EF depends on specific cognitive profiles, focusing on verbal comprehension, perceptual reasoning, and working memory, as well as potential sex differences in EF and intelligence across both groups. Based on these objectives, the study addressed the following research questions:

1. Is intelligence associated with executive-function capacity in adolescents with ASD?
2. Do adolescents with ASD differ from their neurotypical peers—matched for age and IQ—in EF performance and in three IQ factors (verbal comprehension, perceptual reasoning, working memory)?
3. Are there sex-related differences in EF, IQ profiles, or autistic traits within the sample?

## Methods

### Measures of executive functions

The executive functions were assessed using the Wisconsin Card Sorting Test (WCST) and the Color Trails Test (CTT). For participants aged 12–16 years old, intelligence was measured with the Wechsler Intelligence Scale for Children-Revised (WISC-R), while for older participants, the Wechsler Adult Intelligence Scale-Revised (WAIS-R) was administered. Table 1 presents the executive function tests along with their descriptions.

**Table 1:** Description of the tests of executive functions used in the study.

Test	Description	Measurement
Wisconsin Card Sorting Test (WCST)	The WCST is designed to assess the efficiency of working memory, as well as the level of development of the following executive functions: planning, rule making and application, attention switching, and cognitive flexibility (Heaton et al., 2008). During the test, the participant is presented with a deck of cards that varies in the number, color, and shape of the symbols. The task is to sort these cards according to a classification rule determined by the examiner. The sorting rule is not disclosed to the participant and is changed periodically throughout the task without warning, requiring the participant to detect the new rule based on feedback. In the Polish adaptation, reliability was assessed using indicators of absolute stability—namely, the correlations between scores obtained in the individual test categories across two measurement points spaced over time—as well as the concordance among expert raters. In the Polish adaptation, the correlation coefficients for absolute stability ranged from 0.42 to 0.85, depending on the category, whereas inter-rater agreement was very high (Kendall’s W, rounded, equaled 1.0) (Jaworska, 2002).	Executive functions were measured using the following variables from the WCST: (1) Planning – represented by the percentage of conceptual responses, the number of completed/achieved categories, and the total number of correct responses; (2) Flexibility – represented by the percentage of perseverative errors and the percentage of perseverative responses. Additional WCST measures included: learning efficiency, the number of non-perseverative errors, the percentage of non-perseverative errors, and the total number of errors.
The Color Connection Test (CTT)	The CTT is a neuropsychological tool designed to assess attention, processing speed, cognitive flexibility, and executive control. The test consists of two parts. In CTT-1, participants are required to use a pencil to connect numbered circles printed in alternating colors (yellow and pink) in ascending numerical order. In CTT-2, the task is similar but requires alternating between colors while following the numerical sequence, which increases executive demands by engaging set-shifting and divided attention. The Polish adaptation (Łojek and Stańczak, 2012) demonstrated satisfactory reliability, with Pearson’s correlation coefficients for performance time between the first and subsequent measurements of *r* = 0.54 for CTT-1 and *r* = 0.86 for CTT-2. For the interference index, the concordance of interpretation by competent judges was *t* = 0.62.	Executive functions were measured using the following variables from the CTT: (1) Switching disruption rate – calculated as the difference between the completion times for CTT-2 and CTT-1, relative to the CTT-1 time; this index reflects attentional metastability, divided attention, and inhibitory control; (2) CTT-2 completion time – an indicator of attentional metastability and cognitive switching efficiency. Additional CTT measures included: CTT-1 completion time, indicating visual search efficiency. All analyses in the present study were based on raw scores.

### Wechsler intelligence scale for children-revised (WISC-R)

The WISC-R, commonly used to assess general, crystallized, and fluid intelligence in children aged 6 to 16 years and 11 months, was applied to measure the cognitive functioning of adolescents aged 12 to 16 years and 11 months. The Polish adaptation (Ciarkowska et al., 2012) demonstrates high psychometric properties, with Cronbach’s alpha coefficients ranging from 0.73 to 0.96 for the entire scale and from 0.76 to 0.87 for its subscales.

### Wechsler adult intelligence scale-revised (WAIS-R)

The WAIS-R, designed to assess general, crystallized, and fluid intelligence in individuals aged 17 years and older, was used to measure the cognitive functioning of participants aged 17 and above. The Polish adaptation (Brzeziński et al., 2004) demonstrates satisfactory to high internal consistency, with reliability coefficients calculated using the Tellegen and Briggs formula ranging from 0.52 to 0.90 for individual subtests and from 0.80 to 0.90 for the subscales.

### Wechsler intelligence factors

The analysis focused not only on full-scale IQ but also on specific cognitive factors derived from individual subtests of the WISC-R and WAIS-R. First, the results of the relevant subtests were averaged to compute composite scores representing distinct cognitive domains (Brzeziński et al., 2004; Ciarkowska et al., 2012). This procedure yielded the following factors:

- Verbal Comprehension—based on scores from Information, Vocabulary, Comprehension, and Similarities subtests,
- Perceptual Reasoning—based on Picture Completion, Picture Arrangement, Block Design, and Object Assembly,
- Working Memory and Resistance to Distractors—based on Arithmetic, Digit Span, and Digit Symbol.

These factors were used in further analyses to examine group differences and associations with EF measures.

### Autism quotient (AQ)

The AQ questionnaire, available in adolescent and over-16 versions (Baron-Cohen et al., 2006), is used to assess everyday, social, and emotional functioning and to estimate the severity of autistic traits. We applied this questionnaire to control severity in autistic traits both in group with psychiatric diagnosis of ASD as well in the group without such diagnosis. It consists of 50 statements grouped into five subscales: Social Skill, Communication, Imagination, Attention Switching and Attention to Detail. For participants under 16 years of age, the questionnaire is completed by parents, whereas for those aged 16 and above, the self-report version is used. The Polish self-report adaptation demonstrates acceptable reliability, with Cronbach’s alpha coefficients ranging from 0.45 to 0.71 for the subscales, and from 0.75 (typically developing adults) to 0.86 (individuals with ASD) for the total score (Pisula et al., 2013). Responses are given on a four-point scale without a neutral option (*Completely agree*, *Rather agree*, *Rather disagree*, *Completely disagree*).

### Participants and procedures

The initial pool consisted of 330 adolescents who participated in a broader research project. Inclusion in the ASD group was based on a clinical diagnosis of Autism Spectrum Disorder, confirmed according to the International Classification of Diseases (ICD-10). From this group, a final sample of 214 participants was selected for the present analysis. This sample included 118 adolescents with ASD (42 girls and 76 boys) and 96 typically developing (TD) counterparts. Participants were match on full-scale IQ and chronological age. There were no significant differences between the ASD and TD groups in terms of age or IQ. Detailed characteristics of the final sample are presented in Table 2.

**Table 2:** Characteristics of the sample.

Characteristic	ASD group			Control group		
	(*N* = 118)	Girls (*n* = 42)	Boys (*n* = 76)	(*N* = 96)	Girls (*n* = 33)	Boys (*n* = 63)
Age M (years)	14	13.77	14.22	14.49	13.73	14.89
SD	2.52	2.70	2.41	2.47	2.37	2.41
Full scale IQ (M)	106.42	106.43	106.41	106.75	103.24	108.59
SD	14.98	15.79	14.62	12.19	10.47	12.69
AQ total scores (M)	24.73	23.98	25.15	16.41	18.08	15.38
SD	9.97	7.76	11.04	8.69	9.16	8.33

AQ, Autism Spectrum Quotient; SD, standard deviation; M, mean.

Adolescents with ASD were recruited through diagnostic centers and schools, whereas TD participants were recruited exclusively from schools. All assessments were conducted by trained researchers in separate, quiet rooms located in diagnostic and therapy clinics or within school settings. During the first meeting, participants completed tests of executive functions and other cognitive measures that were part of the broader project. The Wechsler Intelligence Scale was administered during the second meeting. The study received ethical approval from the Ethics Committee of the Faculty of Psychology at the University of Warsaw, Poland (the opinion number 6/11/2022).

### IQ-based group classification

Based on the normative classification from the Polish standardization of the WISC-R, where an IQ of 111 represents the threshold for above-average intellectual functioning, four groups were distinguished according to full scale IQ level and diagnostic status:

- ASD High-IQ group (*n* = 41, M = 123.56, IQ 111–140),
- ASD Low-IQ group (*n* = 78, M = 97.3, IQ 85–110),
- Control High-IQ group (*n* = 28, M = 121.86 IQ 112–140),
- Control Low-IQ group (*n* = 68, M = 100.53, IQ 85–110).

#### Autism symptom severity (AQ scores)

**Figure 1:**
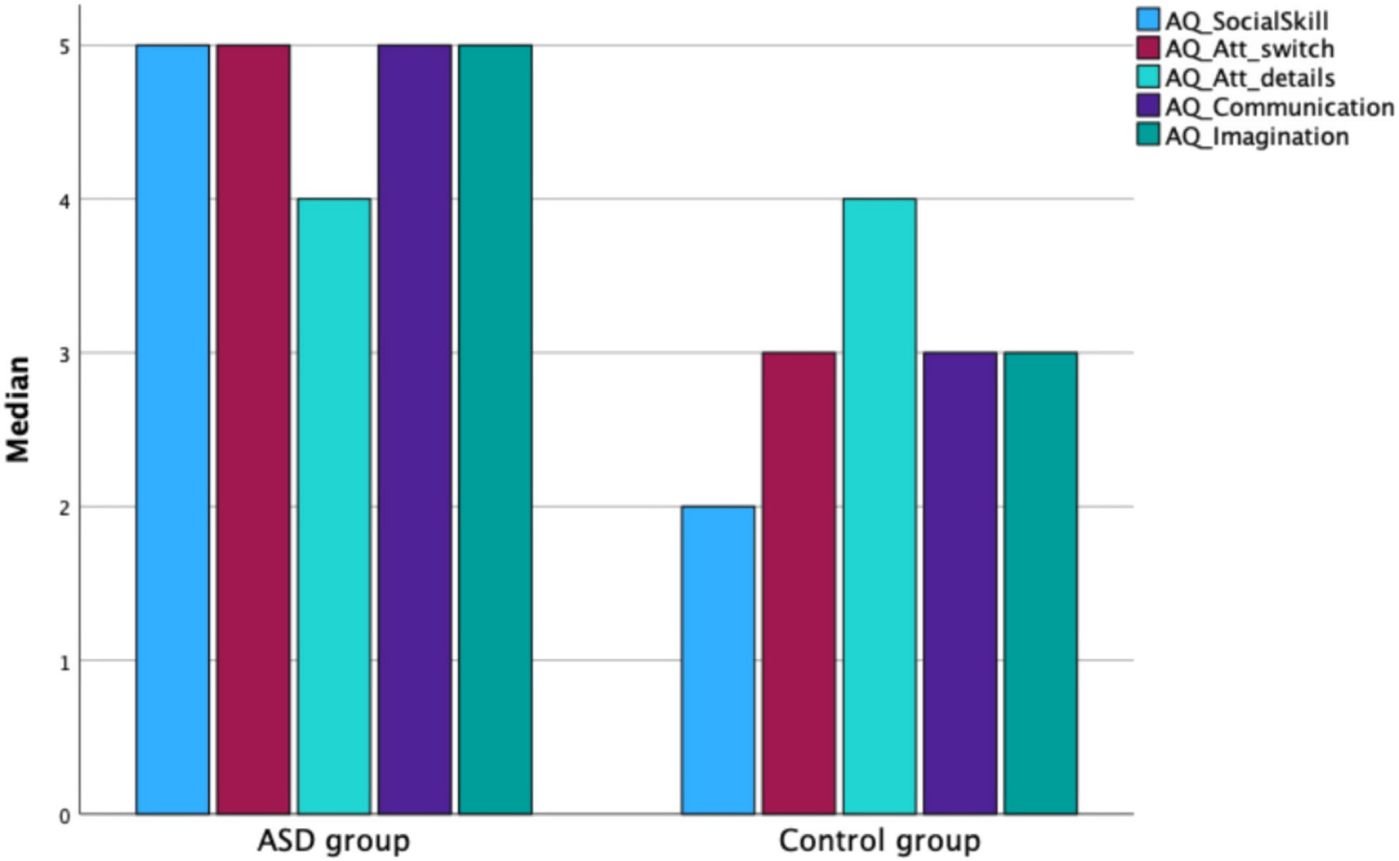
Median scores for AQ subscales in the ASD and control groups.

Mann–Whitney U test results showed that adolescents with ASD obtained significantly higher total scores on the Autism Spectrum Quotient (AQ) compared to their neurotypical peers (*Z* = −5.908, *p* < 0.001). The ASD group had a higher median AQ score (Mdn = 25; IQR = 8) than the control group (Mdn = 17.5; IQR = 9.75). Statistically significant group differences were also found for most AQ subscales: Social Skills (*Z* = −7.306, *p* < 0.01), Attention Switching (*Z* = −3.674, *p* < 0.001), Communication (*Z* = −3.992, *p* < 0.001), and Imagination (*Z* = −3.893, *p* < 0.001). In all these domains, the ASD group scored significantly higher than the control group (see Figure 1).

#### Sex differences in AQ scores

**Figure 2:**
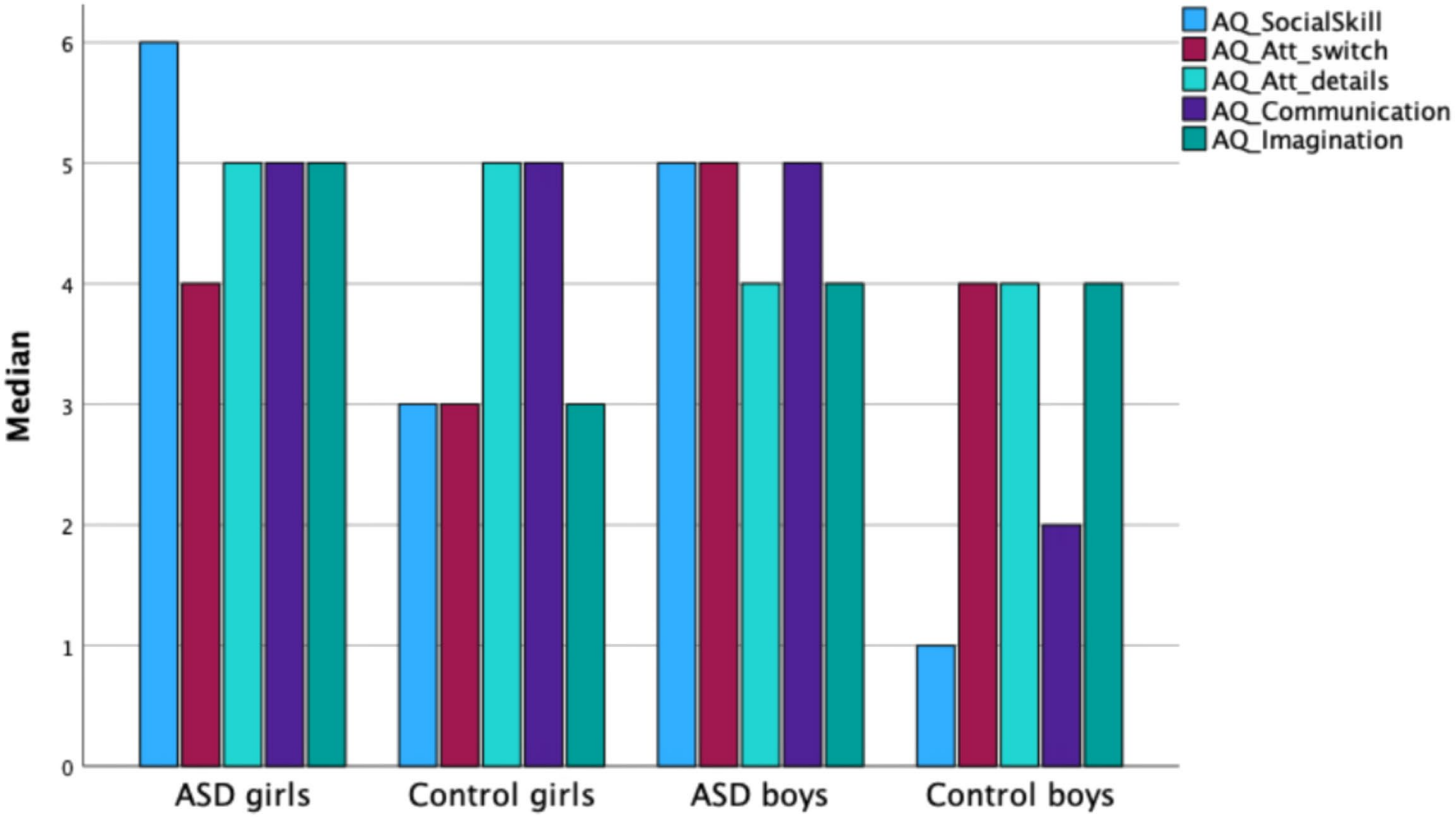
Median AQ subscale scores for girls and boys in the ASD group and in the control group.

Mann–Whitney U test results showed a significant difference between boys and girls in Attention Switching (*Z* = −3.074, *p* < 0.01), with boys scoring higher than girls (see Figure 2). Within the female subsample, significant differences were found between girls with ASD and girls in the control group for the AQ total score and the following subscales: Social Skills (*Z* = −4.449, *p* < 0.001), Attention Switching (*Z* = −2.054, *p* < 0.04), and Imagination (*Z* = −2.830, *p* < 0.01). Girls with ASD scored significantly higher than control-group girls on these measures, while no significant group differences were observed in Communication. Within the male subsample, significant differences were found between boys with ASD and control-group boys for the AQ total score and four subscales: Social Skills (*Z* = −5.563, *p* < 0.001), Attention Switching (*Z* = −3.280, *p* < 0.001), Communication (*Z* = −4.486, *p* < 0.001), and Imagination (*Z* = −2.594, *p* < 0.01). Boys with ASD scored significantly higher than control-group boys on all these measures.

## Results

Given that the distribution of several variables significantly deviated from normality in both the clinical group (ASD) and the control group, non-parametric Mann–Whitney U tests were employed for between-group comparisons. This approach was chosen to ensure robust statistical inference despite the non-normal distribution of data, which is common in studies involving clinical populations. The results of the Kolmogorow-Smirnow test are presented in Appendix 2 across four comparisons related to EF analyses (ASD vs. Control Group, four groups regarding IQ low&high IQ, sex differences boys vs. girls in full sample and sex differences within ASD and control group).

### Performance on the WCST and CTT

#### ASD vs. control group

Mann–Whitney U test results indicated no statistically significant differences between the ASD group (N = 118) and the control group (N = 96) in planning and flexibility (WCST), or switching (CTT). No significant group differences were observed for Switching Time (CTT-2), Switching (Interference Index), or Visual Search (CTT-1).

#### ASD group with low IQ vs. ASD group with high IQ

Mann–Whitney U test results showed that the ASD group with low IQ performed less efficiently than the ASD group with high IQ on flexibility measures: percentage of perseverative responses (Z = −3.49, *p* < 0.001) and percentage of perseverative errors (Z = −3.68, *p* < 0.001), as well as on planning measures: percentage of conceptual responses (Z = −2.51, *p* < 0.05) and number of achieved categories (Z = −2.54, *p* < 0.011) and in switching (Z = −2.143, *p* < 0.05) (see Table 3).

**Table 3:** Comparison between the ASD group with low IQ and the ASD group with high IQ in planning, flexibility, and switching measures (Mann–Whitney U test).

Variables	Median – ASD Low-IQ group	Median – ASD High-IQ group	Mann–Whitney U test	*Z*	*p*-value
Planning (WCST)					
Total correct responses	71	70	1,422	−0.57	0.056
Percentage of conceptual responses	61	75.5	920	−2.515	0.012
Number of completed/achieved categories	6	6	1,150	−2.65	0.008
Flexibility (WCST)					
Percentage of perseverative errors	15	9	886	−3.84	0.001
Percentage of perseverative responses	17	10	920	−3.65	0.001
Switching (CTT)					
Disruption rate	0.84	0.77	1,451	−0.721	0.471
CTT-2 completion time	48.5	41	1199.5	−2.143	0.032

#### Control group with high IQ vs. control group with low IQ

Mann–Whitney U test results revealed that individuals in the control group with high IQ completed the CTT-2 (switching) significantly faster than their peers with low IQ (*Z* = −2.762, *p* < 0.005). In one planning measure—the number of achieved categories—controls with high IQ also scored higher than those with low IQ (*Z* = −2.613, *p* < 0.01). No significant differences were found between these groups in the number of conceptual responses or total correct responses. The groups also did not differ significantly in flexibility measures (see Table 4).

**Table 4:** Comparison of WCST and CTT performance between the control group with low IQ and the control group with high IQ across planning, flexibility, and switching measures (Mann–Whitney U test).

Variables	Median – control group (low IQ)	Median – control group (high IQ)	Mann–Whitney U test	*Z*	*p*-value
Planning (WCST)					
Total correct responses	70	69	866.500	−0.013	0.990
Percentage of conceptual responses	68	76.5	659.500	−1.818	0.069
Number of completed/achieved categories	4	6	638.500	−2.613	0.009
Flexibility (WCST)					
Percentage of perseverative errors	12	9.5	696.00	−1.504	0.133
Percentage of perseverative responses	12.95	10.5	668.000	−1.747	0.081
Switching (CTT)					
Disruption rate	0.841	0.774	862.5	−0.722	0.471
CTT-2 completion time	48	41	609.5	−2.762	0.006

#### ASD group with high IQ vs. control group with high IQ

Mann–Whitney U test results showed no statistically significant differences between the ASD group with high IQ and the control group with high IQ in planning, flexibility, or switching measures (see Table 5).

**Table 5:** Comparison of WCST and CTT performance between the ASD group with high IQ and the control group with high IQ across planning, flexibility, and switching measures (Mann–Whitney U test).

Variable	Median – ASD high IQ	Median – control high IQ	Mann–Whitney U test	*Z*	*p*-value
Planning (WCST)					
Total correct responses	70	69	567.50	−0.080	0.937
Percentage of conceptual responses	75.50	76.50	525	−0.611	0.541
Number of completed/achieved categories	6	6	529	−0.990	0.322
Flexibility (WCST)					
Percentage of perseverative errors	9	9.50	501	−0.895	0.371
Percentage of perseverative responses	10	10.50	509	−0.797	0.426
Switching (CTT)					
Disruption rate	0.774	1.060	436	−1.686	0.092
CTT-2 completion time	41	38.75	478	−1.174	0.240

#### ASD group with low IQ vs. control group with low IQ

**Figure 3:**
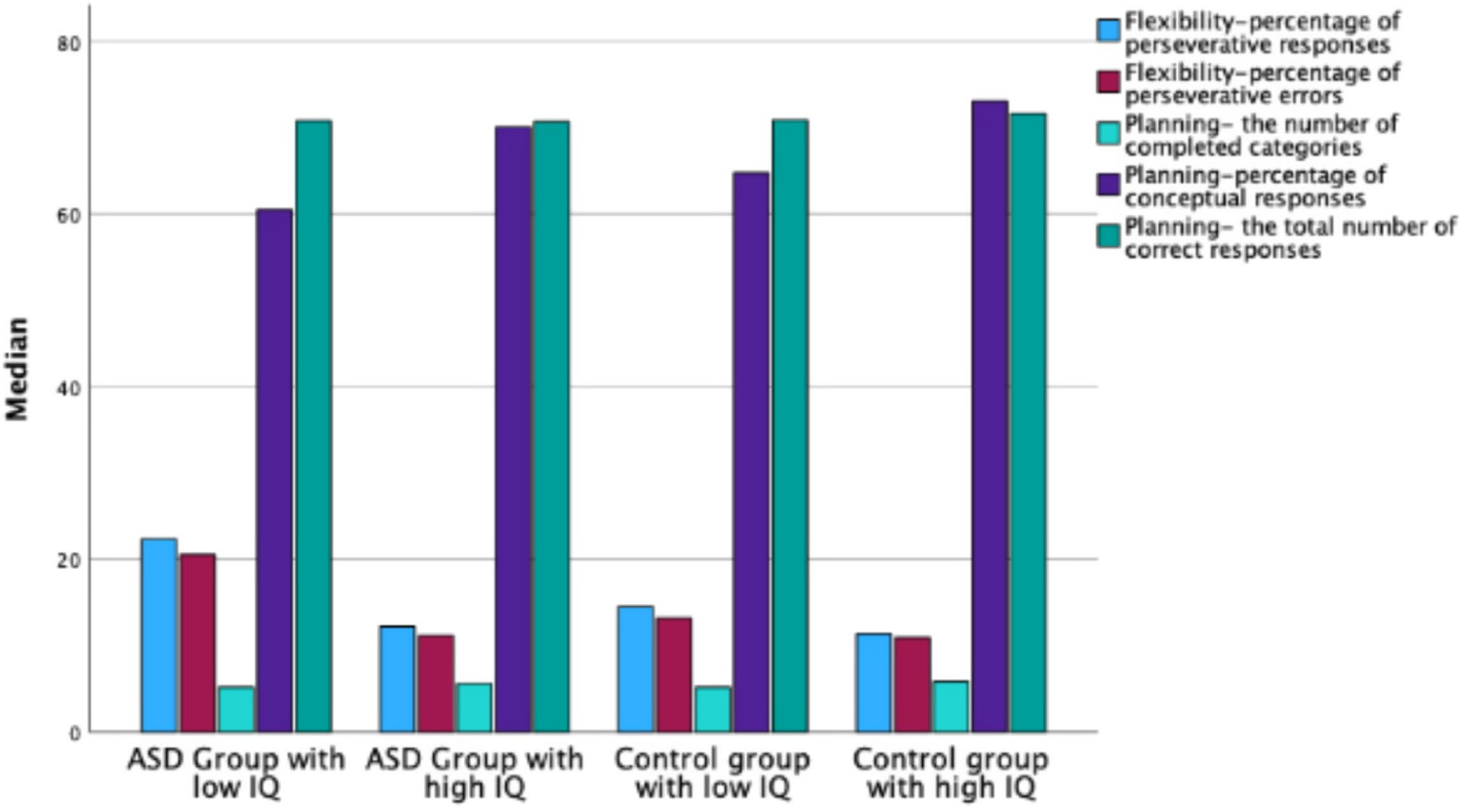
Median WCST scores in ASD groups with low and high IQ and in control groups with low and high IQ.

Mann–Whitney U test results showed significant differences between the ASD group with low IQ and the control group with low IQ in *flexibility* measures: percentage of perseverative errors (*Z* = −2.529, *p* < 0.05) and percentage of perseverative responses (*Z* = −2.254, *p* < 0.05) (see Figure 3). No significant differences were observed between these groups in *planning* or *switching* measures (see Table 6).

**Table 6:** Comparison of WCST and CTT performance between the ASD group with low IQ and the control group with low IQ across planning, flexibility, and switching measures (Mann–Whitney U test).

Variable	Median – ASD low IQ	Median – control low IQ	Mann–Whitney U test	*Z*	*p*-value
Planning (WCST)					
Total correct responses	71	70	2,244	−0.353	0.724
Percentage of conceptual responses	61	75.5	1988	−1.340	0.180
Number of completed/achieved categories	6	6	2,120	−1.002	0.316
Flexibility (WCST)					
Percentage of perseverative errors	15	9	1,692	−2.529	0.011
Percentage of perseverative responses	17	10	1830	−2.254	0.024
Switching (CTT)					
Disruption rate	0.841	1.00	2412.50	−0.814	0.416
CTT-2 completion time	48.50	50	2567.50	−0.202	0.840

### Performance on the WCST and CTT, and sex differences

#### Girls vs. boys

Mann–Whitney U test results indicated no statistically significant differences between girls and boys in any of the planning or flexibility measures from the WCST, nor any of the measures from the CTT.

#### ASD group girls vs. control group girls

Mann–Whitney U test results indicated no statistically significant differences between girls with ASD and girls in the control group in any of the planning or flexibility measures from the WCST, nor in switching measures from the CTT.

#### ASD group boys vs. control group boys

Mann–Whitney U test results indicated a significant difference in planning—number of completed categories (*Z* = −2.137, *p* < 0.05)—with boys from the control group scoring higher (Mean Rank = 76.06) than boys from the ASD group (Mean Rank = 63.99). No significant differences were found between the groups in flexibility measures or in switching measures from the CTT (disruption rate and CTT-2 completion time).

#### ASD group girls vs. ASD group boys

Mann–Whitney U test results indicated no statistically significant differences between boys and girls within the ASD group in any of the WCST measures.

### Performance on the IQ factors

The analyses included three main cognitive factors derived from WISC-R and WAIS-R subtests: Verbal Comprehension, Perceptual Reasoning, Working Memory and Resistance to Distractors.

#### ASD vs. control group

Mann–Whitney U test results indicated no statistically significant differences between the ASD and control groups in Verbal Comprehension, Perceptual Reasoning, or Working Memory and Resistance to Distractors.

#### ASD group with low IQ vs. ASD group with high IQ

There were no significant differences between these groups in any of the IQ factors, indicating that the high-IQ and lower-IQ groups did not differ in their intelligence profiles, with both remaining within the normal range of intelligence.

### Control group with high IQ vs. control group with low IQ

For the IQ factors, the control group with high IQ achieved significantly higher scores than the control group with low IQ in all three domains: Perceptual Reasoning (*Z* = −5.359, *p* < 0.001), Verbal Comprehension (*Z* = −5.387, *p* < 0.001), and Working Memory/Resistance to Distractors (*Z* = −4.958, *p* < 0.001).

#### ASD group with high IQ vs. control group with high IQ

**Figure 4:**
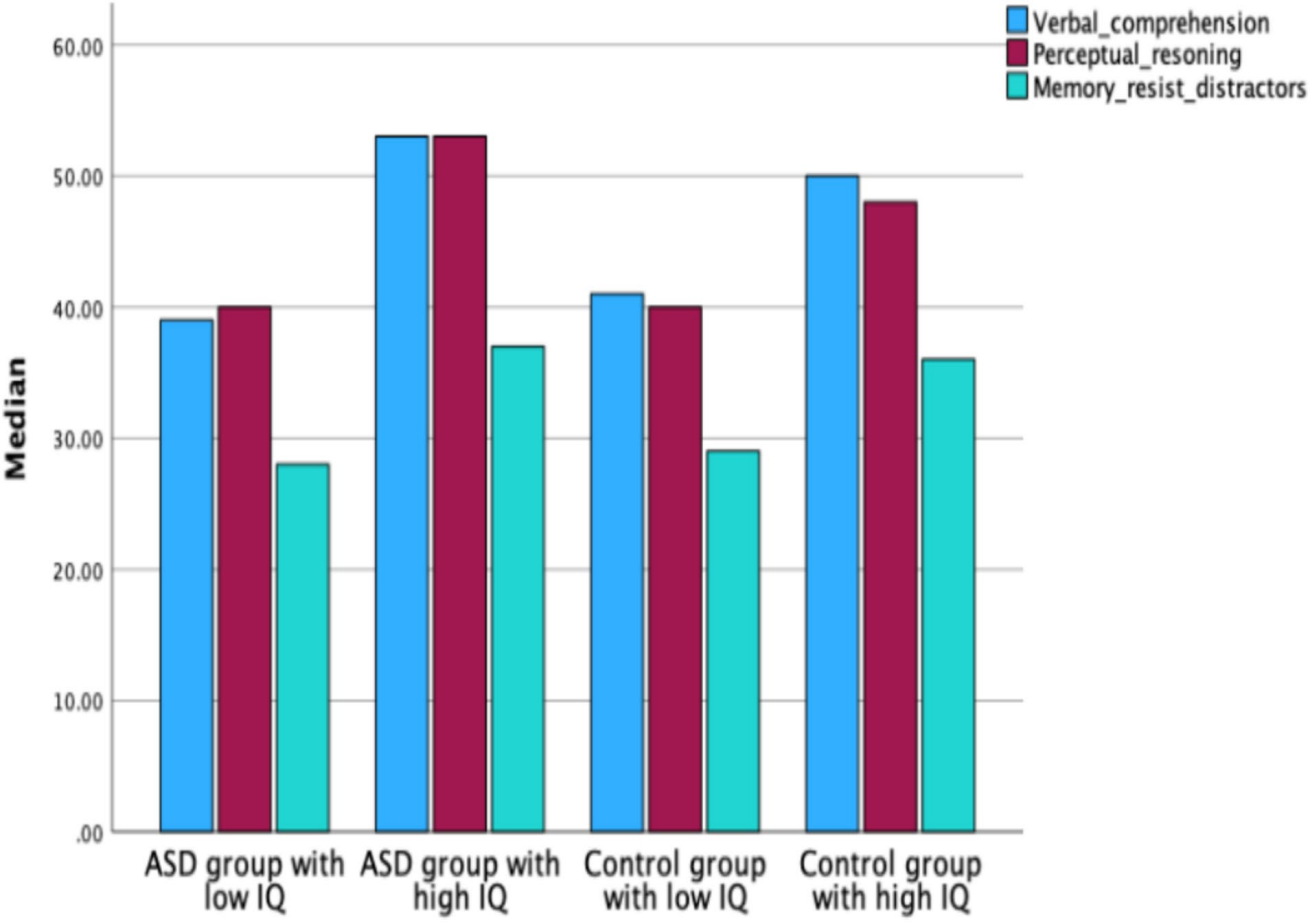
Median scores for IQ factors: verbal comprehension, perceptual reasoning, and working memory/resistance to distractors, in ASD groups with low and high IQ and control groups with low and high IQ.

The ASD group with high IQ scored significantly higher (Mdn = 54) than the control group with high IQ (Mdn = 41) in the IQ factor Verbal Comprehension (*Z* = −2.136, *p* < 0.001) (see Figure 4).

### Sex differences in IQ factors

**Figure 5:**
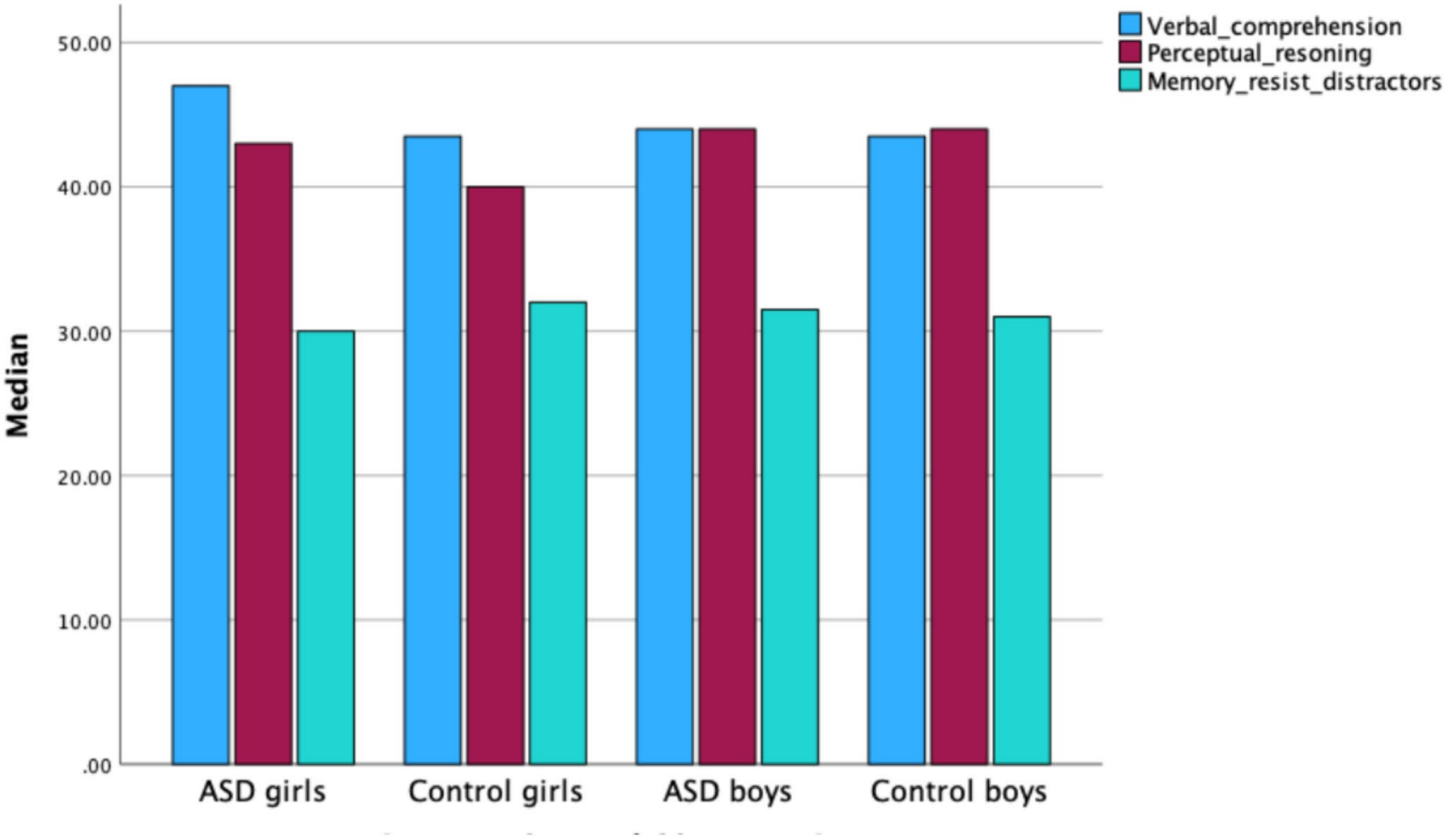
Median scores for IQ factors—verbal comprehension, perceptual reasoning, and working memory/resistance to distractors—in boys and girls across the ASD and control groups.

No significant sex differences were found between all boys and all girls in Perceptual Reasoning (*Z* = −2.078, *p* < 0.05). Within the control group, boys scored significantly higher than girls in Perceptual Reasoning (*Z* = −2.545, *p* < 0.05). No significant differences were observed between boys and girls within the ASD group (see Figure 5).

### The relationship of EF with intelligence

Presented below are the correlations with Spearman’s rho coefficients above 0.30 between IQ scales (full, verbal, and non-verbal) and IQ factors (Verbal Comprehension, Perceptual Reasoning, Working Memory/Resistance to Distractors) with WCST and CTT measures. The descriptive statistics in WCST, CTT and IQ factors are presented in Supplementary materials (Appendix 1).

#### ASD group

The Spearman correlation analysis in the ASD group revealed a weak negative correlation between full-scale IQ and Flexibility, measured as the percentage of perseverative responses (*ρ* = −0.341, *p* < 0.001) and the percentage of perseverative errors (ρ = 0.357, *p* < 0.001), as well as with Switching, measured by CTT-2 completion time (ρ = −0.358, *p* < 0.001). Non-verbal IQ showed moderate correlations with Switching (CTT-2 completion time; ρ = −0.464, *p* < 0.001), Planning (percentage of conceptual responses; ρ = 0.312, *p* < 0.001), and Flexibility (percentage of perseverative errors; ρ = −0.421, *p* < 0.001). Among IQ factors, Perceptual Reasoning was significantly correlated with Flexibility (percentage of perseverative responses; ρ = −0.362, *p* < 0.001; percentage of perseverative errors; ρ = −0.391, *p* < 0.001) and Switching (CTT-2 completion time; ρ = −0.418, *p* < 0.001).

#### Control group

Planning, measured by the number of completed categories, was positively correlated with full-scale IQ (ρ = 0.325, *p* < 0.01), non-verbal IQ (ρ = 0.306, *p* < 0.01), and the IQ factor Perceptual Reasoning (ρ = 0.329, *p* < 0.01). In addition, the IQ factor Memory/Resistance to Distractors was positively correlated with another Planning indicator—the percentage of conceptual responses (ρ = 0.322, *p* < 0.001). Both the non-verbal IQ scale and the IQ factor Memory/Resistance to Distractors were negatively correlated with Switching, measured as CTT-2 completion time (ρ = −0.311, *p* < 0.01).

## Discussion

The present study examined the relationship between intelligence and EF with Autism Spectrum Disorder without intellectual disability, taking into account IQ level, sex, and comparisons with typically developing peers matched for age and full-scale IQ. Contrary to initial expectations, adolescents with ASD did not differ significantly from the control group in EF performance on standardized measures, such as the Wisconsin Card Sorting Test and the Color Trails Test. This finding supports previous observations that structured, decontextualized neuropsychological tasks may fail to capture the subtle executive difficulties experienced by individuals with ASD=WID in everyday life (Barkley, 2012; Kenworthy et al., 2008). This previously observed pattern confirms that when cognitive ability is controlled, EF performance in ASD-WID may approximate that of typically developing youth in laboratory conditions (Rašková et al., 2025).

However, a more nuanced analysis revealed that within the ASD group, intelligence level was meaningfully associated with EF performance. Adolescents with higher IQs showed significantly better outcomes in cognitive flexibility and planning (WCST) and in the completion time in CTT, but not in the disruption rate A similar pattern was found in the control group, where individuals with higher IQ completed the CTT-2 more efficiently and achieved more categories in the WCST. These findings are consistent with previous research suggesting that intelligence contributes to executive control processes, particularly in tasks that require abstraction, strategy generation, and rule inference (Cruz et al., 2022; Dawson et al., 2007; Friedman et al., 2006).

Importantly, no group differences emerged in EF performance between adolescents with ASD and controls within the high-IQ subgroup, suggesting that cognitive capacity may buffer executive weaknesses in autism. This constitutes a new contribution, indicating that high cognitive abilities may reduce observable EF differences between ASD and TD adolescents (Livingston and Happé, 2017; Mottron et al., 2006). In contrast, adolescents with ASD and lower IQ exhibited more perseverative errors than neurotypical peers with similar IQ levels, highlighting a clearer additive effect of autistic features and limited cognitive resources on executive control. These results align with prior findings (Geurts et al., 2004; Kenworthy et al., 2008; Landa and Goldberg, 2005) but extend them by demonstrating that such EF differences become particularly pronounced when ASD co-occurs with lower intellectual functioning.

Additionally, the analysis of IQ components revealed no significant differences between the ASD and control groups in verbal comprehension, perceptual reasoning, or working memory and resistance to distractors. This replicated pattern suggests broadly similar cognitive profiles when full-scale IQ is matched, but the present findings refine earlier reports by emphasizing within-group variability in ASD-WID.

The results contrast with earlier reports of uneven IQ profiles in ASD (e.g., elevated performance IQ or verbal IQ discrepancies) (Chiang and Gau, 2016; Planche and Lemonnier, 2012). Notably, cognitive strengths and weaknesses in ASD-WID may not be uniformly distributed, but may vary depending on IQ level and task demands.

Sex differences also emerged in the broader sample. Boys across the full cohort (ASD+ control) demonstrated significantly higher scores in perceptual reasoning than girls, consistent with well-established sex-related advantages in visuospatial processing in the general population (Linn and Petersen, 1985; Halpern et al., 2007). However, these differences were not observed within the ASD group providing new evidence that autism may attenuate or obscure typical sex-linked cognitive patterns (Lai et al., 2015; Lai and Szatmari, 2020). It is possible that autistic girls and boys share more similar developmental trajectories in domains such as non-verbal reasoning, or that the unique neurocognitive phenotype of females with ASD masks typical sex differences observed in neurotypical populations (Lai and Szatmari, 2020; Zhang et al., 2024). These findings underscore the importance of investigating sex as a moderator of cognitive performance within both clinical and non-clinical populations.

Taken together, the results of this study highlight the significant role of intelligence—particularly non-verbal reasoning—in modulating executive functioning outcomes in adolescents with and without ASD. By distinguishing findings that replicate prior evidence (e.g., typical EF performance on structured tasks, associations between IQ and EF) from new observations (compensation in high-IQ ASD, additive deficits in low-IQ ASD, attenuation of sex differences), the overall interpretation becomes more coherent and nuanced. Moreover, the findings indicate that while high-functioning individuals with ASD may perform similarly to their neurotypical peers on standardized EF tasks, those with lower cognitive resources may experience increased EF difficulties. This underscores the need for individualized assessments sensitive to specific cognitive strengths and weaknesses.

The present findings highlight the importance of cognitive profile in performing EF tasks. In particular, associations between perceptual reasoning, non-verbal IQ, and EF markers suggest that interventions may benefit from leveraging visuospatial strengths. Strategies based on visual schemas, mind maps, or step-by-step instructions may enhance planning and organizational skills in this group. More operational intervention approaches include the use of graphic organizers, visual task sequences, step-by-step visual scaffolding, and graded rule-shifting or set-shifting exercises, which may be particularly beneficial for adolescents with lower IQ.

The lack of differences between the high-IQ ASD group and their neurotypical counterparts may reflect the phenomenon of *camouflaging*—the use of learned compensatory strategies that enable performance within the normative range in laboratory conditions. However, such compensation may not generalize to real-life contexts with higher cognitive or social load, indicating the need for supports aimed at reducing executive demands. It is also important to note that the WCST and CTT have limited ecological validity, as they lack emotional and social components that strongly influence EF in daily functioning. Adolescence is a period of significant structural changes in the prefrontal cortex, which can promote both plasticity and compensation and may increase variability in EF performance. Therefore, longitudinal research is needed to determine whether the observed patterns persist across development.

### Limitations and future directions

Several limitations should be considered when interpreting these findings. First, although the WCST and CTT are well-established EF measures, they may not fully capture the complexity of everyday executive functioning; performance-based tasks often underestimate real-world difficulties, particularly in behavioral regulation and cognitive control in social contexts (Barkley, 2012; Kenworthy et al., 2008). Future research should therefore include more ecologically valid tools (e.g., parent/teacher questionnaires, naturalistic tasks). Second, the cross-sectional design limits conclusions about developmental trajectories and causality. Longitudinal studies are needed to clarify whether higher IQ provides lasting cognitive resilience or whether EF difficulties intensify as environmental demands increase. Third, despite careful matching for age and IQ, other influential factors—such as socioeconomic status, comorbidities (e.g., ADHD, anxiety), and educational support—were not controlled and should be addressed in future work. Fourth, the relatively small number of girls with ASD may have reduced power to detect subtle sex effects; larger, sex-balanced samples are needed to deepen understanding of sex-specific cognitive profiles and compensatory strategies. Finally, the exclusive focus on adolescents limits generalizability. Studies including younger children and adults are necessary to determine whether similar patterns persist across development and to inform age-appropriate interventions.

Despite these limitations, the study provides both replicated and novel insights into how intelligence modulates EF in ASD-WID, highlighting the importance of cognitively informed, individualized intervention strategies.

## Data Availability

The raw data supporting the conclusions of this article will be made available by the authors, without undue reservation.
