# Supplementary material for: Intelligence and executive functioning in adolescence: comparing autism spectrum disorder and typical development
Source: Front Psychol. 2026 Jan 7;16:1733356. doi: 10.3389/fpsyg.2025.1733356 (PMC12819240; doi:10.3389/fpsyg.2025.1733356)
Supplement: Supplementary file 1 [file Supplementary_file_1.docx]

Appendix 1

Descriptive statistics in Wisconsin Sorting Card Test (WCST) and Colour Trails Test (CTT) in ASD and control group

|  | ASD | | |  | Control group | | | |
| --- | --- | --- | --- | --- | --- | --- | --- | --- |
| Variables | Minimum | Maximum | M | SD | Minimum | Maximum | M | SD |
| Planning (WCST) | | | | | | | | |
| Total correct responses | 21 | 98 | 70.48 | 11.841 | 36 | 97 | 71.08 | 11.84 |
| Percentage of conceptual responses | 5.00 | 99.00 | 63.79 | 19.89 | 9.00 | 90.00 | 67.34 | 16.64 |
| Number of achived categories | 0 | 6 | 46874,00 | 2.18 | 0 | 6 | 13636 | 1.26 |
| Flexiblity (WCST) | | | | | | | | |
| Percentage of perseverative errors | 3.00 | 111.00 | 17.27 | 16.13 | 4.00 | 43.00 | 937914 | 6.08 |
| Percentage of perseverative responses | 3.00 | 106.00 | 18.90 | 17.92 | 4.00 | 57.00 | 13.51 | 7.48 |
| Switching (CTT) | | | | | | | | |
| Disruption rate | -0,96 | 13971 | .90 | .68 | -.39 | 2.78 | 45748 | .66 |
| CTT-2 completion time | 1.00 | 240.00 | 54.18 | 28.74 | 20 | 138 | 52.08 | 23.51 |

Note: M: mean; SD: standard deviation.

Appendix 2

Descriptive statistics in IQ scales and IQ factors and in ASD and control group

|  | ASD group | | | | Control group | | | |
| --- | --- | --- | --- | --- | --- | --- | --- | --- |
| Variables | Minimum | Maximum | M | SD | Minimum | Maximum | M | SD |
| full scale IQ | 85 | 144 | 106.42 | 14.98 | 86 | 144 | 106.75 | 12.191 |
| IQ verbal scale | 69 | 147 | 106.26 | 16.86 | 78 | 151 | 107.99 | 14.587 |
| IQ non-verbal scale | 72 | 151 | 105.32 | 16.08 | 77 | 141 | 104.11 | 13.357 |
| Verbal comprehension | 14.00 | 72.00 | 43.54 | 11.70 | 25.00 | 63.00 | 43.4792 | 8.25129 |
| Perceptual resoning | 23.00 | 70.00 | 43.90 | 9.95 | 24.00 | 64.00 | 42.6250 | 8.38200 |
| Memory/ Resistance to Distractors | 10.00 | 50.00 | 30.97 | 9.15 | 17.00 | 46.00 | 31.27 | 6.09143 |

Abbreviation: M: mean; SD: standard deviation
