# Supplementary material for: Intelligence and executive functioning in adolescence: comparing autism spectrum disorder and typical development
Source: Front Psychol. 2026 Jan 7;16:1733356. doi: 10.3389/fpsyg.2025.1733356 (PMC12819240; doi:10.3389/fpsyg.2025.1733356)
Supplement: Supplementary file 2 [file Supplementary_file_2.docx]

Appendix 3

Table A

Kolmogorow-Smirnow results of normal distribution in WCST and CTT variables in ASD group and Control Group

|  |  | D | df | p |
| --- | --- | --- | --- | --- |
| WCST |  |  |  |  |
| Total correct responses | ASD group | .132 | 115 | <.001 |
|  | Control group | .094 | 90 | .046 |
| Percentage of conceptualresponses | ASD group | .113 | 115 | <.001 |
|  | Control group | .144 | 90 | <.001 |
| Number of achieved categories | ASD group | .357 | 115 | <.001 |
|  |  |  |  |  |
|  | Control group | .437 | 90 | <.001 |
|  |  |  |  |  |
| Percentage of perseverative errors | ASD group | .127 | 115 | <.001 |
|  |  |  |  |  |
|  | Control group | .170 | 90 | <.001 |
|  |  |  |  |  |
| Percentage of perseverative responses | ASD group | .200 | 115 | <.001 |
|  | Control group | .174 | 90 | <.001 |
| CTT |  |  |  |  |
| Disruption rate | ASD group | .104 | 115 | .004 |
|  | Control group | .092 | 90 | .056 |
| CTT-2 completion time | ASD group | .146 | 115 | <.001 |

Control group .151 90 <.001

Table B

Kolmogorow-Smirnow results of normal distribution in WCST and CTT variables in ASD group (low & high IQ)and Control Group (low &high IQ)

| Variables | Group | D | df | p |
| --- | --- | --- | --- | --- |
| WCST |  |  |  |  |
| Total correct responses | ASD group low IQ | 0.141 | 74 | <.001 |
|  | ASD highIQ | 0.163 | 41 | 0.008 |
|  | Control group low IQ | 0.09 | 62 | .200* |
|  | Control group high IQ | 0.138 | 28 | 0.184 |
| Percentage of conceptual responses | ASD group low IQ | 0.086 | 74 | .200* |
|  | ASD group high IQ | 0.177 | 41 | 0.002 |
|  | Control group low IQ | 0.123 | 62 | 0.022 |
|  | Control group high IQ | 0.169 | 28 | 0.038 |
| Number of achieved categories | ASD group low IQ | 0.344 | 74 | <.001 |
|  | ASD highIQ | 0.504 | 41 | <.001 |
|  | Control group low IQ | 0.386 | 62 | <.001 |
|  | Control group high IQ | 0.534 | 28 | <.001 |
| Percentage of perseverative errors | ASD group low IQ | 0.093 | 74 | 0.18 |
|  | ASD highIQ | 0.209 | 41 | <.001 |
|  | Control group low IQ | 0.124 | 62 | 0.02 |
|  | Control group high IQ | 0.196 | 28 | 0.008 |
| Percentage of perseverative responses | ASD group low IQ | 0.2 | 74 | <.001 |
|  | ASD highIQ | 0.19 | 41 | <.001 |
|  | Control group low IQ | 0.168 | 62 | <.001 |
|  | Control group high IQ | 0.154 | 28 | 0.089 |
| CTT |  |  |  |  |
| Disruption rate | ASD group low IQ | 0.127 | 74 | 0.005 |
|  | ASD highIQ | 0.108 | 41 | .200* |
|  | Control group low IQ | 0.099 | 62 | .200* |
|  | Control group high IQ | 0.132 | 28 | .200* |
| CTT-2 completion time | ASD group low IQ | 0.167 | 74 | <.001 |
|  | ASD highIQ | 0.168 | 41 | 0.005 |
|  | Control group low IQ | 0.124 | 62 | 0.019 |
|  | Control group high IQ | 0.19 | 28 | 0.011 |

Table C

Kolmogorow-Smirnow results of normal distribution in WCST and CTT variables in boys and girls (full sample).

| Variables | groups | D | df | p |
| --- | --- | --- | --- | --- |
| WCST |  |  |  |  |
| Total correct responses | ASD group | .132 | 115 | <.001 |
|  | Control group | .094 | 90 | .046 |
| Percentage of conceptualresponses | ASD group | .113 | 115 | <.001 |
|  | Control group | .144 | 90 | <.001 |
| Number of achieved categories | ASD group | .357 | 115 | <.001 |
|  |  |  |  |  |
|  | Control group | .437 | 90 | <.001 |
|  |  |  |  |  |
| Percentage of perseverative errors | ASD group | .127 | 115 | <.001 |
|  | Control group | .170 | 90 | <.001 |
|  |  |  |  |  |
| Percentage of perseverative responses | ASD group | .200 | 115 | <.001 |
|  | Control group | .174 | 90 | <.001 |
| CTT |  |  |  |  |
| Disruption rate | ASD group | .104 | 115 | .004 |
|  | Control group | .092 | 90 | .056 |
| CTT-2 completion time | ASD group | .146 | 115 | <.001 |
|  | Control group | .151 | 90 | <.001 |

Table D

Kolmogorow-Smirnow results of normal distribution in WCST and CTT variables in girls with ASD and girls control group, boys with ASD and boys control group

| Variables | Groups | D | df | p |
| --- | --- | --- | --- | --- |
| WCST |  |  |  |  |
| Total correct responses | Girls ASD | 0.134 | 41 | 0.062 |
|  | Girls control group | 0.094 | 27 | .200* |
|  | Boys ASD | 0.13 | 74 | 0.003 |
|  | Boys control group | 0.123 | 63 | 0.019 |
| Percentage of conceptual responses | Girls ASD | 0.121 | 41 | 0.136 |
|  | Girls control group | 0.213 | 27 | 0.003 |
|  | Boys ASD | 0.137 | 74 | 0.002 |
|  | Boys control group | 0.134 | 63 | 0.007 |
| Number of achieved categories | Girls ASD | 0.465 | 41 | <.001 |
|  | Girls control group | 0.365 | 27 | <.001 |
|  | Boys ASD | 0.364 | 74 | <.001 |
|  | Boys control group | 0.471 | 63 | <.001 |
| Percentage of perseverative errors | Girls ASD | 0.108 | 41 | .200* |
|  | Girls control group | 0.219 | 27 | 0.002 |
|  | Boys ASD | 0.139 | 74 | 0.001 |
|  | Boys control group | 0.143 | 63 | 0.003 |
| Percentage of perseverative responses | Girls ASD | 0.25 | 41 | <.001 |
|  | Girls control group | 0.215 | 27 | 0.003 |
|  | Boys ASD | 0.196 | 74 | <.001 |
|  | Boys control group | 0.146 | 63 | 0.002 |
| CTT |  |  |  |  |
| Disruption rate | Girls ASD | 0.122 | 41 | 0.126 |
|  | Girls control group | 0.123 | 27 | .200* |
|  | Boys ASD | 0.118 | 74 | 0.012 |
|  | Boys control group | 0.107 | 63 | 0.072 |
| CTT-2 completion time | Girls ASD | 0.152 | 41 | 0.018 |
|  | Girls control group | 0.194 | 27 | 0.011 |
|  | Boys ASD | 0.178 | 74 | <.001 |
|  | Boys control group | 0.139 | 63 | 0.004 |
